# Supplementary material for: Physicians’ satisfaction with clinical laboratory services at public hospitals in Ethiopia: A national survey
Source: PLoS One. 2020 Apr 30;15(4):e0232178. doi: 10.1371/journal.pone.0232178 (PMC7192407; doi:10.1371/journal.pone.0232178)
Supplement: S1 Data — (DOCX) [file pone.0232178.s001.docx]

**Region:__________ Town:___________ Zone___________ Health Facility:______________**

| **Part IV: Socio-Demographic Characteristics of Physicians** | | | | | |
| --- | --- | --- | --- | --- | --- |
| **S.No** | **Questionnaires** | | | **Responses Classification** |  |
| 401 | Sex of the respondents | | | Male….1 Female…..2 |  |
| 402 | Age in years | | | ______years |  |
| 403 | What is your marital status? | | | Single….…....1 Married………2  Divorced…….3 Widowed……4 |  |
| 404 | What is your educational level? | | | MD-----------------1  Specialized-----------2 specify ______________ |  |
| 405 | How many years of experience do you have? | | | __________years |  |
| 406 | Your current work station/ward? | | | ___________ |  |
| **Part V:Physician’s Satisfaction with Clinical Laboratory Services** | | | | | |
| **S.No** | | **Questionnaires** | **Responses Classification** | |  |
| 501 | | Do you have updated laboratory hand book? | Yes……..1  No………2 | |  |
| 502 | | How much are you satisfied with the assistance of the hand book to utilize the lab services? | Very satisfied………….1  Satisfied……………….2  Neutral…………………3  Dissatisfied…………….4  Very dissatisfied……….5 | |  |
| 503 | | Are laboratory personnel available when you attempt to contact them? | Yes…………1  No………….2 | |  |
| 504 | | How much are you satisfied with timely expert/advisory service of lab staff at times of need (directly or through telephone and email communications)? | Very satisfied………….1  Satisfied……………….2  Neutral…………………3  Dissatisfied…………….4  Very dissatisfied……….5 | |  |
| 505 | | How much are you satisfied with the laboratory’s ability to resolve complaints? | Very satisfied………….1  Satisfied……………….2  Neutral…………………3  Dissatisfied…………….4  Very dissatisfied……….5 | |  |
| 506 | | How much are you satisfied with the laboratory’s request form? | Very satisfied………….1  Satisfied……………….2  Neutral…………………3  Dissatisfied…………….4  Very dissatisfied……….5 | |  |
| 507 | | How much are you satisfied with the test menu of the laboratory to manage your patient? | Very satisfied………….1  Satisfied……………….2  Neutral…………………3  Dissatisfied…………….4  Very dissatisfied……….5 | |  |
| 508 | | If no (4, 5), does the lab have a backup or referral service system? | Yes……………1  No…………….2 | |  |
| 509 | | If yes, are you comfortable with the backup or referral lab test reports? | Yes …………..1  No……………2 | |  |
| 510 | | Does the laboratory post turnaround time of available tests in your work area? | Yes………….1  No…………...2 | |  |
| 511 | | Do you receive the laboratory report within the agreed turnaround time? | Yes…………..1  No……………2 | |  |
| 512 | | How much are you satisfied with clarity, legibility and completeness of laboratory report? | Very satisfied………….1  Satisfied……………….2  Neutral…………………3  Dissatisfied…………….4  Very dissatisfied……….5 | |  |
| 513 | | How much are you satisfied with the provision of STAT (urgent/rush) services in a timely fashion? | Very satisfied………….1  Satisfied……………….2  Neutral…………………3  Dissatisfied…………….4  Very dissatisfied……….5 | |  |
| 514 | | Does the laboratory immediately notify panic results/ (Critical value notification) | Yes…………1  No………….2 | |  |
| 515 | | Does the laboratory provide appropriate notification to you when new test technologies are deployed? | Always …………1  Sometimes..…….2  No………………3 | |  |
| 516 | | Does the laboratory notify to you when there is test interruption on time? | Always …………1  Sometimes..…….2  No………………3 | |  |
| 517 | | How much do you satisfied with the lab-clinical interface? | Very satisfied………….1  Satisfied……………….2  Neutral…………………3  Dissatisfied…………….4  Very dissatisfied……….5 | |  |
| 518 | | Do you feel the quality of work is consistent with all shifts (working hour, weekend, holiday, night duty) | Yes…………1  No………….2 | |  |
| 519 | | In which shift, the quality of lab service has been compromised? | _____________________ | |  |
| 520 | | How much are you satisfied with overall laboratory services? | Very satisfied………….1  Satisfied……………….2  Neutral…………………3  Dissatisfied…………….4  Very dissatisfied……….5 | |  |
